# HOIL-1L Interacting Protein (HOIP) Is Essential for CD40 Signaling

**DOI:** 10.1371/journal.pone.0023061

**Published:** 2011-08-01

**Authors:** Bruce S. Hostager, Masaki Kashiwada, John D. Colgan, Paul B. Rothman

**Affiliations:** Department of Internal Medicine, University of Iowa, Iowa City, Iowa, United States of America; University Freiburg, Germany

## Abstract

CD40 is a cell surface receptor important in the activation of antigen-presenting cells during immune responses. In macrophages and dendritic cells, engagement of CD40 by its ligand CD154 provides signals critical for anti-microbial and T cell-mediated immune responses, respectively. In B cells, CD40 signaling has a major role in regulating cell proliferation, antibody production, and memory B cell development. CD40 engagement results in the formation of a receptor-associated complex that mediates activation of NF-κB, stress-activated protein kinases, and other signaling molecules. However, the mechanisms that link CD40 to these signaling events have been only partially characterized. Known components of the CD40 signaling complex include members of the TNF receptor-associated factor (TRAF) family of proteins. We previously showed that the TRAF family member TRAF2 mediates recruitment of HOIL-1L-interacting protein (HOIP) to the cytoplasmic domain of CD40, suggesting that HOIP has a role in the CD40 signaling pathway. To determine the role of HOIP in CD40 signaling, we used somatic cell gene targeting to generate mouse B cell lines deficient in HOIP. We found that the CD40-induced upregulation of CD80 and activation of germline immunoglobulin epsilon transcription were defective in HOIP-deficient cells. We also found that the CD40-mediated activation of NF-κB and c-Jun kinase was impaired. Recruitment of IκB kinase proteins to the CD40 signaling complex was undetectable in HOIP-deficient cells, potentially explaining the defect in NF-κB activation. Restoration of HOIP expression reversed the defects in cellular activation and signaling. These results reveal HOIP as a key component of the CD40 signaling pathway.

## Introduction

CD40 signaling in professional antigen-presenting cells, including B cells, macrophages, and dendritic cells, is critical for the efficient activation of humoral and cell-mediated immune responses [1], [2], [3]. CD40 signaling is activated in a T cell-dependent manner, as the ligand for CD40, CD154, is expressed primarily by activated T cells. CD40 engagement leads to the activation of various signaling molecules, including stress-activated protein kinases and the transcription factor NF-κB, which upregulate the expression of cytokines and other factors that promote immune responses. The mechanism by which CD40 induces these signaling pathways has not been completely defined. The cytoplasmic domain of CD40 does not appear to have intrinsic enzymatic activity, but is able to mediate signaling through the recruitment of several intracellular proteins. Members of the TNF receptor-associated factor (TRAF) family, including TRAF1, TRAF2, TRAF3, and TRAF6, appear to be particularly important for the initiation and regulation of CD40 signaling [4]. These proteins function in part as adaptor molecules, binding to the cytoplasmic tail of CD40 and recruiting other proteins to the receptor-associated complex. Some of the TRAFs also function as E3 ubiquitin ligases, and this enzymatic activity may contribute to signal propagation and regulation. Among the multiple TRAFs that associate with CD40, TRAF3 can function as a negative regulator of signaling, while TRAF2 and TRAF6 promote the activation of downstream signaling pathways [4].

We recently demonstrated that HOIL-1L interacting protein (HOIP), a ubiquitin ligase that can catalyze the assembly of linear polyubiquitin chains [5], is recruited to CD40 in a TRAF2-dependent manner following engagement of CD40 by agonistic antibody [6]. These and other findings led us to hypothesize that HOIP functions downstream of TRAF2 in the CD40 signaling pathway and that HOIP is necessary for the activation of NF-κB and possibly other signaling molecules. To test this hypothesis, we employed somatic cell gene targeting to ablate expression of HOIP in a mouse B cell line that has proven to be a useful model for B cell CD40 signaling [7], [8], [9]. We found that the CD40-induced upregulation of CD80 (a costimulatory molecule for T cells) was defective in HOIP-deficient cells. Similarly, the CD40 and IL-4 driven production of germline transcripts from the immunoglobulin epsilon heavy chain locus, an event that precedes immunoglobulin gene rearrangement and isotype switching, was defective in the absence of HOIP. We also found that the CD40-mediated activation of NF-κB and the stress-activated protein kinase c-Jun kinase (JNK) was defective in HOIP-deficient cells. Consistent with impaired NF-κB activation, association of the IκB kinase (IKK) complex with CD40 was undetectable in HOIP-deficient cells. Together, our results indicate that HOIP plays a critical role in the activation of signaling pathways that regulate cellular responses to CD40 engagement.

## Results

### Generation of HOIP-deficient B cells via targeted disruption of *Rnf31*, the gene encoding HOIP

**Figure 1:**
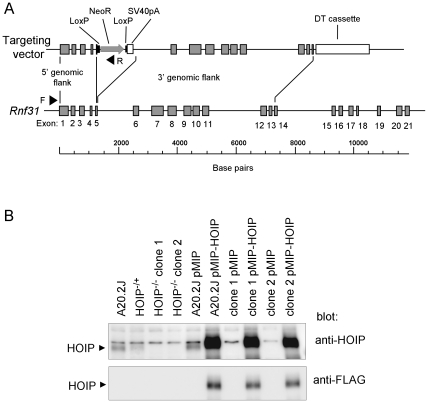
HOIP gene targeting. (A) Regions of *Rnf31* gene sequence (bottom) used in the HOIP gene targeting vector (top) are shown. Arrows (F and R) indicate approximate positions of sequences homologous to oligonucleotides used for PCR-mediated detection of homologous recombination. Homologous recombination of the vector with *Rnf31* resulted in a small chromosomal deletion and the in-frame insertion of neomycin phosphotransferase (NeoR) into the amino-terminal HOIP coding sequence. A diphtheria toxin (DT) cassette in the vector facilitates the negative selection of cells in which random chromosomal insertion of the vector takes place. LoxP sequences allow the Cre-mediated deletion of the NeoR coding sequence. The SV40pA sequence helps to ensure disruption of gene expression after deletion of the NeoR sequence. (B) Anti-HOIP and anti-FLAG Western blots of cell lysates from A20.2J cells and HOIP-deficient (HOIP^-/-^) clones. A partial decrease in HOIP protein expression is evident in cells following disruption of one copy of *Rnf31* (HOIP^−/+^). HOIP expression in clones reconstituted with an empty retroviral vector (pMIP) or a retroviral vector encoding FLAG-tagged HOIP is also shown. Approximate molecular weight of HOIP is 120 kD.

In our previous study, we demonstrated that HOIP is recruited to the CD40 signaling complex in two mouse B cell lines [6]. Similar results were obtained with mouse splenocytes, indicating that the interaction of CD40 with HOIP is not limited to transformed B cell lines (unpublished observations). We also found that the recruitment of HOIP to CD40 was TRAF2-dependent and that overexpression of a truncated HOIP mutant partially inhibited CD40-mediated NF-κB activation. These results support the hypothesis that HOIP plays a role in CD40 signal transduction. To further define the role and evaluate the importance of HOIP in CD40 signaling, we used somatic cell gene targeting to disrupt the gene encoding HOIP in the mouse B cell line A20.2J. This cell line has been particularly useful in the characterization of CD40 signaling mechanisms due to the relative ease with which its genome can be modified by homologous recombination [7], [8], [9]. We used a targeting vector capable of undergoing homologous recombination with *Rnf31* (the gene encoding HOIP) to disrupt the coding sequence of the gene in exon 5 (Fig. 1A). Following introduction of the vector, the neomycin-resistant clones that arose were screened by PCR amplification of genomic DNA to identify cells containing a disrupted *Rnf31* allele. To remove the selectable marker gene cassette from the disrupted *Rnf31* allele, recombinant cell lines were transiently transfected with a plasmid that encodes Cre recombinase. This step allowed us to perform a second round of targeting and drug selection, generating cells in which both copies of *Rnf31* were disrupted. Two independent clonal cell lines were chosen for further analysis. HOIP protein expression was undetectable in both cell lines, as determined by Western blot analysis of cell lysates (Fig. 1B), demonstrating that the targeting process was successful. In the text that follows, the two gene-targeted cell lines are referred to as HOIP-deficient cells.

To enable us to confirm that any signaling or functional defects observed in HOIP-deficient cells were due to disruption of the *Rnf31* gene, both HOIP-deficient cell lines were transduced with a retrovirus encoding FLAG-tagged wild-type HOIP, thus restoring HOIP protein expression (Fig. 1B). These cells are referred to as HOIP-reconstituted cells. In the experiments that follow, responses by these cells were compared to those of A20.2J cells and HOIP-deficient cells transduced with a retroviral vector (pMIP) lacking a cDNA insert (empty vector).

### HOIP is required for CD40-mediated CD80 upregulation and activation of germline epsilon transcription

**Figure 2:**
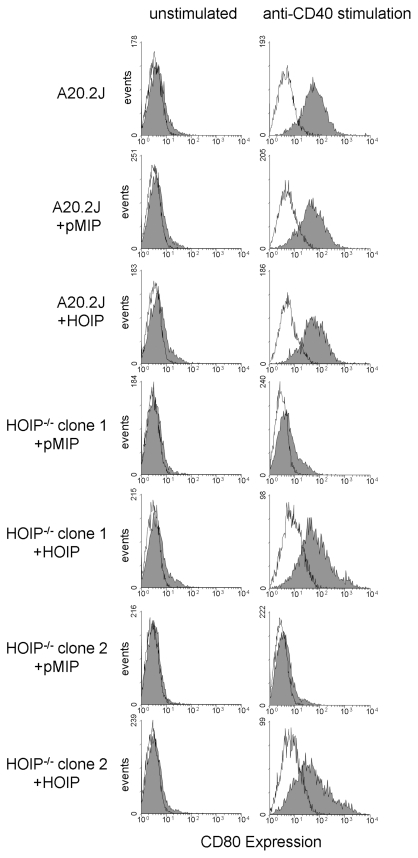
CD40-mediated CD80 upregulation is defective in HOIP-deficient cells. Cells were incubated for 72 hrs with an isotype control antibody (left) or anti-CD40 antibody (right) and then stained for expression of CD80 (filled profiles indicate staining with anti-CD80 antibody; open profiles are staining with an isotype control antibody). Transduction of both HOIP-deficient (HOIP^-/-^) clones with a retrovirus encoding FLAG-HOIP reversed defective CD80 upregulation, while transduction with an empty vector (pMIP) did not. Similar results were obtained in a second experiment.

Engagement of CD40 on B cells upregulates expression of CD80 [10], a cell surface protein that promotes activation of T cells interacting with B cells and other APC. To begin evaluating potential contributions of HOIP to CD40 signaling, we assayed the upregulation of CD80 induced by engagement of CD40 on parental A20.2J, HOIP-deficient, and HOIP-reconstituted cells (Fig. 2). Compared to the parental cell line, the CD40-stimulated upregulation of CD80 was dramatically reduced in all HOIP-deficient cells tested. In contrast, upregulation of CD80 was restored in HOIP-reconstituted cells, confirming that the defect in CD80 upregulation in the HOIP-deficient cells was due to the disruption of *Rnf31*.

**Figure 3:**
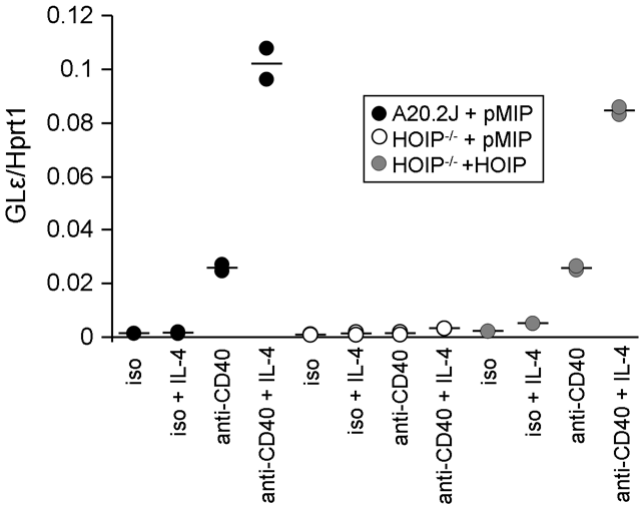
GLε transcription is defective in CD40-stimulated HOIP-deficient cells. A20.2J and HOIP-deficient cells transduced with an empty retroviral vector (A20.2J + pMIP and HOIP^-/-^ + pMIP, respectively), or HOIP-deficient cells transduced with a HOIP-encoding retrovirus (HOIP^-/-^ + HOIP) were cultured overnight with agonistic anti-CD40 or an isotype control antibody (iso), with or without IL-4. RNA was isolated from the cells, reverse-transcribed, and then subjected to quantitative PCR to determine levels of GLε transcripts. Results were normalized to the levels of Hprt1 transcripts in each sample. Symbols indicate the values from duplicate cultures (a line indicates the mean of the two values). Similar results were obtained in a second experiment and in an additional experiment with a second HOIP-deficient clone.

CD40 signals in B cells can also contribute to the activation of DNA recombination in the immunoglobulin heavy chain locus [11], [12]. This process, known as immunoglobulin class switching (or isotype switching), allows B cells, initially expressing IgM, to switch to IgA, IgG, or IgE depending on the class of antibody most appropriate for a particular immune response. Switching to IgE can be induced by the combination of IL-4 and CD40 signals. The gene rearrangement necessary for IgE production is preceded by the production of non-coding RNA transcripts from germline sequences in the IgE heavy chain locus [13]. To determine whether HOIP is required in the initiation of the class switching process, the relevant B cell lines were stimulated through CD40 in the presence of IL-4 and tested for production of germline epsilon (GLε) transcripts (Fig. 3). Anti-CD40 antibody stimulation alone or together with IL-4 induced GLε transcription in parental A20.2J cells as expected. In contrast, only low levels of GLε expression were detected in HOIP-deficient cells stimulated with either anti-CD40 antibody alone or anti-CD40 and IL-4. Normal levels of GLε expression were induced in HOIP-reconstituted cells stimulated with anti-CD40 antibody alone or together with IL-4. Collectively, these data demonstrate that HOIP has a critical role in cellular responses to CD40 signaling.

### HOIP mediates CD40-stimulated NF-κB and JNK activation

**Figure 4:**
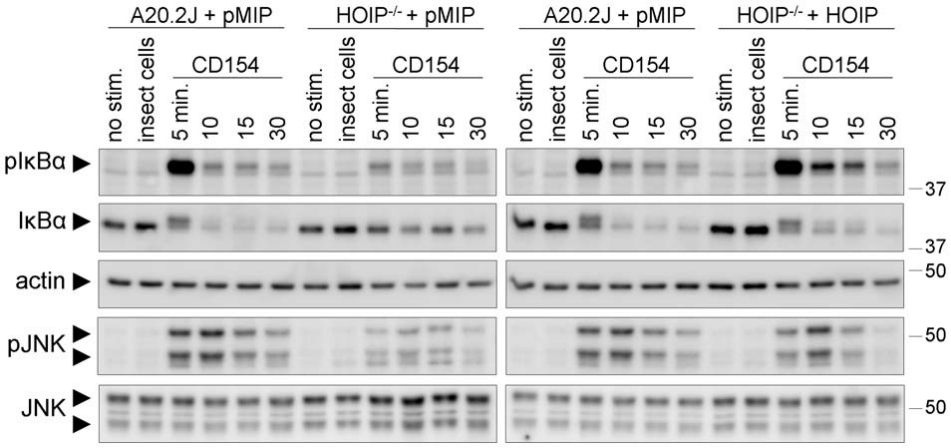
CD40-induced activation of NF-κB and JNK is defective in HOIP-deficient cells. CD40-mediated signaling was defective in HOIP-deficient cells (HOIP^-/-^ + pMIP), but intact in parental cells transduced with an empty retroviral vector (A20.2J + pMIP) and in HOIP-deficient cells transduced with a retroviral vector encoding HOIP (HOIP^-/-^ + HOIP). Cells were activated with CD154-expressing insect cells for the times indicated. As a negative control, cells were stimulated with insect cells lacking CD154 (5 minute time point only). Western blots of whole-cell lysates were probed with the indicated antibodies. Phospho-IκBα (pIκBα) and phospho-JNK (pJNK) blots were stripped and reprobed for total IκBα and total JNK, respectively (anti-JNK antibodies recognize p46 and p54 isoforms). IκBα blots were also reprobed for actin to demonstrate equal lane loading. Molecular weights are indicated at right. Similar results were obtained in a second experiment and in two additional experiments with a second HOIP-deficient clone.

The results described above show that HOIP plays an important role in CD40-mediated effector functions of B cells. It follows that HOIP is likely a key mediator of CD40 signaling. To test this hypothesis, we stimulated A20.2J and HOIP-deficient cells with CD154 (CD40 ligand) expressed by HI5 insect cells [7], [8] and measured activation of the NF-κB and JNK pathways, two of the major transcriptional regulators activated by CD40 [4]. Cell-associated CD154 was used as the stimulus in these experiments as it typically provides more robust, and therefore more readily detected, activation signals than does anti-CD40 antibody. Activation of the canonical NF-κB pathway is initiated with the phosphorylation of IκB proteins by the IκB kinase complex (IKK). In resting cells, IκB proteins are responsible for sequestering NF-κB subunits in the cytoplasm. Phosphorylation by the IKK complex targets IκB proteins for ubiquitination and degradation, allowing NF-κB to enter the nucleus and activate gene expression. CD40-mediated phosphorylation and degradation of IκBα in HOIP-deficient cells was dramatically impaired relative to that observed in parental A20.2J cells (Fig. 4). We also assayed activation of the stress-activated protein kinase JNK in response to CD40 engagement (Fig. 4). CD40-mediated JNK activation in HOIP-deficient cells was impaired as measured by phosphorylation of Thr^183^ and Tyr^185^ in JNK. CD40-induced activation of NF-κB and JNK in HOIP-reconstituted cells was normal, demonstrating that the defects observed in gene-deficient cells were due to the absence of HOIP expression.

### HOIP is necessary for association of the IKK complex with CD40

**Figure 5:**
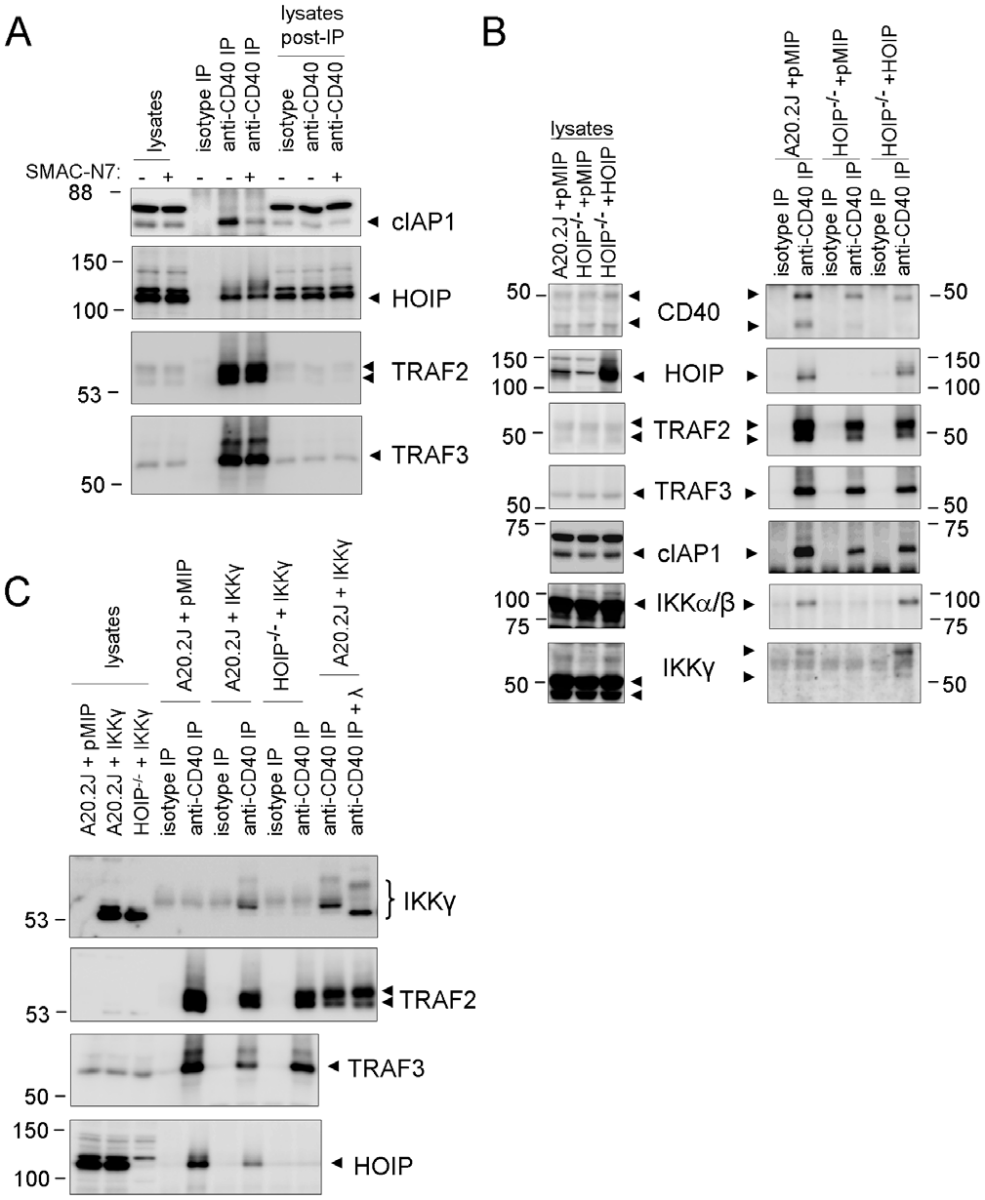
IKK recruitment to the CD40 signaling complex is defective in HOIP-deficient cells. (A) SMAC peptide treatment reduces recruitment of cIAP1 to CD40 and may modify HOIP recruitment. A20.2J cells were incubated for six hours with membrane-permeable SMAC-N7 peptide or 1.5% DMSO (solvent used for the peptide). Following the incubation, cell lysates were prepared, fractionated by SDS-PAGE, and evaluated by Western blot (lanes 1 and 2). Cells incubated with DMSO or SMAC-N7 were also stimulated with magnetic beads coated with anti-CD40 or an isotype control antibody. Immunoprecipitated (IP) material bound to the beads was loaded in lanes 3-5. Samples of the cell lysates after immunoprecipitation appear in lanes 6–8. Western blots were probed with antibodies specific for cIAP1, TRAF2, TRAF3, and HOIP (approximate molecular weights indicated on left). Similar results were obtained in two additional experiments. (B) CD40 was isolated by immunoprecipitation (as in (A)) from A20.2J cells and HOIP-deficient cells transduced with an empty retroviral vector (pMIP) or a retroviral vector encoding HOIP. Material immunoprecipitated with an isotype control antibody (isotype) or anti-CD40 antibody was examined by Western blotting for CD40, TRAF2, TRAF3, cIAP1, HOIP, IKKα/β, and IKKγ (right panels). HOIP expression was required for coprecipitation of IKK proteins with CD40. Cell lysates from unstimulated cells are shown in the left panels. Similar results were obtained in a second experiment and in two experiments with a second HOIP-deficient clone. (C) To further evaluate HOIP-dependent recruitment of IKKγ to CD40, A20.2J cells or HOIP-deficient (HOIP^-/-^) A20.2J cells were transduced with an empty retroviral vector or a retroviral construct encoding BP epitope-tagged IKKγ (noted in the figure as pMIP and IKKγ, respectively). Lysates (lanes 1–3) and immunoprecipitation (IP) samples (lanes 4–11) from the cell lines were fractionated by SDS-PAGE and evaluated by Western blotting with antibodies to the BP tag (IKKγ, upper panel) and TRAF2. The anti-CD40 IP sample in lane 11 was treated with λ phosphatase; the sample in lane 10 was mock-treated. Protein samples (minus those treated with phosphatase) were also fractionated on a separate gel (lower acrylamide concentration) for the evaluation of TRAF3 and HOIP (bottom two panels). Similar results were obtained in two additional experiments.

The marked defects in CD40-mediated cell activation and signaling displayed by HOIP-deficient cells suggested that HOIP mediates recruitment of critical components of the CD40 signaling apparatus to the receptor. Previously, we demonstrated that HOIP is recruited to the CD40 signaling complex in a TRAF2-dependent manner, suggesting that HOIP functions downstream of TRAF2 [6]. Studies by others suggest that the TRAF2-associated proteins cIAP1 and cIAP2 play a role in the recruitment of HOIP to TNFR1 and CD40 [14]. Therefore, we determined whether the association of HOIP with CD40 in A20.2J cells was altered by treatment of cells with an inhibitor of cIAP activity, a membrane-permeable peptide derived from the apoptosis regulator SMAC [15]. We found that pretreatment of cells with the SMAC peptide dramatically reduced the amount of cIAP1 associated with the CD40 signaling complex in cells stimulated with anti-CD40 antibody-coated beads (Fig. 5A). SMAC peptide treatment also resulted in a slight but reproducible decrease in the amount of the major HOIP form recovered by CD40 immunoprecipitation, along with an apparent increase in higher molecular weight species recognized by anti-HOIP antibody. In contrast, treatment with the SMAC peptide did not alter the amount or molecular weight of HOIP present in cell lysates. These data suggest that SMAC peptide treatment specifically alters the characteristics of CD40-associated HOIP rather than the entire cellular pool of this protein. Together, these results support the idea that the cIAP proteins influence the recruitment and post-translational modification state of CD40-associated HOIP.

To test the possibility that HOIP is responsible for the recruitment of other critical signaling proteins to CD40, we immunoprecipitated CD40 signaling complexes from HOIP-deficient and HOIP-reconstituted cells (Fig. 5B). As described previously, CD40 signaling complexes immunoprecipitated from A20.2J cells contained HOIP as well as TRAF2, TRAF3, IKKα/β, and IKKγ [6]. The amounts of TRAF2, TRAF3, and cIAP1 in CD40 immunoprecipitates from HOIP-deficient cells were similar to those from parental A20.2J cells, indicating that HOIP is not required for the association of these proteins with CD40. In contrast, IKKα/β and IKKγ were not detectable in CD40 immunoprecipitates recovered from HOIP-deficient cells (Fig. 5B,C). The amounts of IKKα/β and IKKγ in CD40 immunoprecipitates from HOIP-reconstituted cells were similar to those detected in samples prepared from parental cells, demonstrating that the defects in IKK recruitment we observed were due specifically to the absence of HOIP expression. These data indicate that HOIP is required for recruitment of the NF-κB-activating complex to the CD40 signaling complex.

As shown here and in our previous study [6], IKKγ present in CD40 immunoprecipitates appears to have a higher molecular weight than that found in cell lysates. To confirm that this higher molecular weight species was indeed IKKγ, we generated A20.2J cell lines containing a stably integrated retroviral vector that encoded an epitope-tagged version of mouse IKKγ. Cell lysates and CD40 immunoprecipitates prepared from these cells were analyzed by Western blotting for the epitope tag (Fig. 5C). This analysis produced a pattern of bands that was essentially the same as that obtained using an antibody specific for native IKKγ (compare panels B and C in Fig. 5). Moreover, analysis of cells expressing epitope tagged-IKKγ confirmed that recruitment of IKKγ to CD40 requires HOIP. To determine what was responsible for the increased molecular weight of IKKγ in CD40 immunoprecipitates, the protein samples were incubated with lambda phosphatase, which removes phosphates attached to tyrosine, threonine, or serine residues in proteins. This treatment reduced much of CD40-associated IKKγ to an apparent molecular weight similar to that of the protein in cell lysates (Fig. 5C). However, at least one band of significantly higher molecular weight remained after phosphatase treatment, suggesting that CD40-associated IKKγ is subject to at least one other modification in addition to phosphorylation. Overall, these data demonstrate that HOIP is required for the association of IKKγ with CD40, and suggest that this event is coupled to post-translational modifications of IKKγ.

## Discussion

Our results indicate that the protein HOIP is critical for CD40-induced signals that regulate B cell function. Our data show that HOIP-dependent cellular responses include CD40-mediated upregulation of CD80 expression and synthesis of germline RNA transcripts from the immunoglobulin heavy chain locus, two events that are important for T-cell-dependent antibody-mediated immune responses. At the molecular level, our data indicate that HOIP functions downstream of TRAF2 in the CD40 signaling pathway and that HOIP has a key role in promoting the recruitment of the IKK complex to CD40. Consistent with this, CD40-induced activation of NF-κB is dependent on the presence of HOIP. In addition, our data show that HOIP facilitates the activation of JNK in response to CD40 engagement. Together, our findings provide support for the conclusion that HOIP is a key component of the CD40 signaling pathway. Given the importance of CD40 signaling in both cellular and humoral immune responses, our results indicate that HOIP has a critical role in the regulation of the immune system.

The functional properties of HOIP have only been partially characterized. Initial studies showed that HOIP and the related protein HOIL-1 are components of a large (∼600 kDa) protein complex capable of synthesizing linear polyubiquitin chains [5]. Subsequent studies showed that a HOIP-containing complex can interact with IKKγ and facilitate activation of NF-κB via the canonical pathway [16]. These data, considered together with ours, suggest that a HOIP-containing complex mediates recruitment of IKKγ to the CD40 signaling complex. In addition, CD40-associated HOIP could play a role in activating IKKγ after its recruitment to the signaling complex [16]. The higher molecular weight forms of IKKγ we observed in CD40 immunoprecipitates would be consistent with the presence of post-transcriptional modifications including phosphorylation and ubiquitination, which have been suggested to reduce or enhance IKKγ activity, respectively [16], [17].

The mechanisms by which HOIP mediates recruitment of the IKK complex to CD40 and by which HOIP is recruited to CD40 remain unclear. A previous study indicates that HOIP may mediate direct contacts with the IKK complex [16], suggesting that it functions as an adaptor for the recruitment of the IKK complex to CD40. However, the ubiquitin ligase activity of HOIP suggests that it is more than a simple adapter molecule. As IKKγ appears capable of binding linear polyubiquitin [18], [19], it is possible that HOIP directs formation of linear polyubiquitin chains on a CD40-associated factor, and it is these chains that serve to recruit IKKγ to the CD40 signaling complex. The molecular interactions necessary for recruitment of HOIP itself to the CD40 signaling complex also remain to be fully characterized. We previously showed that the recruitment of HOIP to the signaling complex is TRAF2-dependent [8]. Potentially, TRAF2 and HOIP directly interact, but it is possible that the ubiquitin ligase activity of TRAF2 [20] (or TRAF2-associated proteins, such as the cIAPs [21]) generates K63-linked polyubiquitin chains to which HOIP can bind and thus associate with the CD40 signaling complex [22].

While our previous work indicated a potentially important link between TRAF2 and HOIP in CD40 signaling, the signals and functions tested here are dependent upon TRAF6 as well as TRAF2. In previous experiments with TRAF-deficient A20.2J cells, we found that the activation of NF-κB by CD40 could be mediated by either TRAF2 or TRAF6, while activation of JNK by CD40 was largely dependent on TRAF6 alone [8]. HOIP deficiency compromises the CD40-mediated activation of both NF-κB and JNK, indicating that signals mediated by both TRAF2 and TRAF6 likely pass through HOIP. Our previous work also demonstrated that the CD40-mediated activation of NF-κB and JNK, while TRAF6-dependent, was not compromised by the disruption of the binding site for TRAF6 in the cytoplasmic domain of CD40 or deletion of the receptor binding domain (the TRAF-C domain) in TRAF6 [8]. These observations indicate that TRAF6 need not directly bind CD40 in order to mediate certain signals, suggesting the assembly of a signaling complex not directly associated with the receptor. If such a complex exists, our results indicate that the absence of HOIP compromises its function as well.

Although the experiments presented here focus on CD40, our results and those of other groups [16], [22] support the possibility that HOIP is important in many signaling pathways in which TRAF2 or TRAF6 are involved, including those associated with various members of the TNF receptor superfamily and the Toll-like receptors. The potential importance of HOIP in immune function and TNFR family signaling is further supported by the recent discovery that HOIP interacts with a protein known as SHARPIN (SHANK-associated RH domain interacting protein in postsynaptic density), which appears capable of working together with HOIP and HOIL to mediate the assembly of linear polyubiquitin [14], [23], [24]. Interestingly, mice with a spontaneous mutation in the gene encoding SHARPIN (chronic proliferative dermatitis (cpdm) mice) exhibit chronic inflammation of the skin and internal organs, defective development of secondary lymphoid tissue, and defects in the production of switched immunoglobulin isotypes [17], [25]. The apparently intimate functional link between SHARPIN and HOIP strongly suggests that at least part of the cpdm phenotype stems from defects in the regulation or function of HOIP.

## Materials and Methods

### Cell lines

The mouse B cell line A20.2J has been previously described [26], [27]. Cells were grown in RPMI 1640, 10% FCS, 10 µM 2-ME, 2 mM L-glutamine, and antibiotics. HI-5 insect cells (Invitrogen) expressing CD154 have been described [28].

### Antibodies

Mouse anti-birch profilin antibody (4A6 [29]), rat anti-mouse CD40 (1C10 [30]) and a rat isotype control antibody (mAb72, Developmental Studies Hybridoma Bank, University of Iowa, Iowa City, IA) were isolated from hybridoma supernatants. FITC-labeled anti-mouse CD80 and an isotype control Ab were from eBiosciences. Rabbit anti-HOIP Ab [6] was the kind gift of Dr. Betty Eipper (University of Connecticut Health Center, Farmington, Connecticut). Goat anti-rat IgG, and HRP-labeled secondary Abs were from Jackson ImmunoResearch Laboratories, Inc. All other antibodies used were described previously [6].

### Somatic cell gene targeting

The generation of HOIP-deficient cells was accomplished using a homologous recombination approach described previously [7], [8]. Segments of *Rnf31* gene sequence used in the targeting construct (Fig. 1A) were amplified by PCR from A20.2J genomic DNA. The oligonucleotide primers used to generate the 5′ flank (1224 bp) were 5′-ttttctagagcggtggcttaagtgaccc-3′ and 5′-tattctagatgcagcatctgagaaagcaagc-3′. The 3′ flank (6032 bp) primers were 5′-aaaaccggtgtatgcttctttacgggagaaaaatattag-3′ and 5′-tataccggtatgaagccaaaggaacactgagag-3′. Restriction endonuclease sites in the oligonucleotide primers allowed insertion of the PCR products into the targeting vector. A20.2J cells were transfected (by electroporation [7]) with the targeting construct and subcloned in medium containing 600 µg/ml G418 sulfate. Homologous recombination in G418-resistant clones was detected by PCR of genomic DNA, as described [7]. Oligonucleotide primers used for screening were 5′-cttcctgatctcagctttaccgtcac-3′ (homologous to genomic sequence; approximate position noted in Fig. 1) and 5′-caatccatcttgttcagccat-3′ (homologous to sequence in NeoR). Clones in which one copy of *Rnf31* had been disrupted were transiently transfected with an expression plasmid encoding Cre, in order to remove NeoR. G418-sensitive subclones were subjected to a second round of targeting to disrupt the remaining copy of *Rnf31*. G418-resistant clones were tested for homologous recombination by PCR and HOIP protein expression by Western blot.

### Retroviral transduction

A retroviral vector (pMIP) was used for the stable transduction of cells with HOIP and IKKγ cDNA constructs. pMIP was constructed by replacing the IRES and GFP encoding sequences in pMIG [31] with the IRES and puromycin resistance gene from pIRESpuro2 (Clontech). FLAG-tagged mouse HOIP cDNA was inserted into the multiple cloning site of pMIP. A pMIP construct encoding mouse IKKγ with an amino terminal birch profilin peptide (BP)-tag was also prepared. Retroviral particles were generated by transient transfection of 293T cells with pMIP, pMIP-HOIP, or pMIP-IKKγ and the packaging vector pCL-Eco [32]. Two days after transfection, culture supernatants were harvested and filtered. A20.2J or HOIP-deficient cells were added to 24-well plates (2.5×10^5^ cells/well) with 1 ml virus-containing supernatant and 8 µg/ml polybrene. Plates were centrifuged at 500 x *g* for 2 hrs at room temperature. Cells were cultured in B cell culture medium for 48 hrs, after which the transduced cells were selected in 3 µg/ml puromycin.

### CD80 upregulation and flow cytometry

Cells (5×10^4^ in 2 ml) were cultured in 24-well plates for 3 days with 5 µg/ml anti-CD40 or an isotype control antibody. Cells were stained for flow cytometry with FITC-anti-CD80 or an appropriate control antibody as described [28]. Data acquired with a FACScan flow cytometer (BD Biosciences) were analyzed with WinMDI 2.8 (Scripps Research Institute, San Diego, CA).

### GLε transcript assay

The CD40-simulated activation of GLε transcription was evaluated as previously reported [33]. Briefly, 1×10^6^ cells were stimulated overnight with anti-CD40 antibody (10 µg/ml) or an isotype control antibody, with or without 500 U/ml mouse IL-4 (BD Biosciences). RNA was isolated using Trizol (Invitrogen), and reverse-transcribed (Superscript III kit, Invitrogen). Quantitative PCR for GLε and Hprt1 was performed using SYBR GREEN master mix (Applied Biosystems), and an Applied Biosystems 7900HT Fast Real-Time PCR instrument. Expression of GLε in each sample was normalized to the expression of *Hprt1*.

### NF-κB and JNK activation assays

For activation of CD40 signaling, 5×10^4^ HI5 insect cells or HI5 insect cells expressing mouse CD154 (CD40 ligand) were added to 1×10^6^ B cells. Cells were centrifuged for 1 minute at 400 x *g* (to promote contact between ligand cells and B cells) then incubated at 37°C for times indicated in Fig. 4. Following stimulation, cells were chilled on ice for 2 minutes. Cell pellets were dissolved in 2X SDS-PAGE loading buffer, sonicated, and heated for 5 minutes at 95°C. Total cell lysates (1×10^5^ cell equivalents per lane) were fractionated by SDS-PAGE and blotted to polyvinylidine fluoride (PVDF) membranes. Membranes were probed with antibodies as indicated in Fig. 4. Chemiluminescent detection (Pierce Biotechnology) was used for the visualization of bands on Western blots. Images of blots were recorded with a low-light imaging system (LAS4000, Fuji Medical Systems) and on X-ray film.

### Immunoprecipitations

Immunoprecipitation of CD40 was performed by activated receptor capture, as previously described [6]. Briefly, 3×10^7^ cells (A20.2J and derivatives) were incubated for 60 minutes at room temperature with 10 µl magnetic protein G beads (Dynal) pre-coated with 10 µg goat anti-rat IgG (Jackson), and 10 µg anti-CD40 (1C10) or an isotype control antibody (mAb72). Cells/beads were then pelleted by centrifugation and lysed in buffer containing 1% Triton X100. Beads were washed with lysis buffer to remove unbound material. In some experiments, material associated with the beads was dephosphorylated with lambda phosphatase (New England BioLabs) as per manufacturer's instructions. Beads were resuspended in 2X SDS-PAGE sample buffer and heated for 5 minutes at 95°C. Material eluted from the beads was fractionated by SDS-PAGE and transferred to PVDF membranes for Western blotting. In some experiments, cells were cultured for 6 hrs with 25 µM antennapedia-linked SMAC-N7 peptide (Calbiochem) or an appropriate volume of the solvent used for the peptide (DMSO). After incubation, cells (in peptide- or DMSO-containing medium) were stimulated with antibody-coated beads as outlined above.
